# Role of PUM RNA-Binding Proteins in Cancer

**DOI:** 10.3390/cancers13010129

**Published:** 2021-01-03

**Authors:** Maciej J. Smialek, Erkut Ilaslan, Marcin P. Sajek, Jadwiga Jaruzelska

**Affiliations:** Institute of Human Genetics, Polish Academy of Sciences, Strzeszynska 32, 60-479 Poznan, Poland; erkut.ilaslan@igcz.poznan.pl (E.I.); marcin.sajek@igcz.poznan.pl (M.P.S.)

**Keywords:** cancer, mRNA, PUM proteins, post-transcriptional gene regulation, PTGR, 3′ untranslated regions, 3′UTR

## Abstract

**Simple Summary:**

PUM1 and PUM2 are RNA-binding Pumilio proteins controlling the accessibility of hundreds of mRNAs for translation in a variety of human tissues. As a result, PUMs exemplify one of the mechanisms safeguarding the cellular proteome. PUM expression is disturbed in cancer, resulting in dysregulation of their target mRNAs. These targets encode factors responsible for processes usually affected in cancer, such as proliferation, apoptosis, and the cell cycle. This review describes PUM1 and PUM2 ribonucleoprotein networks and highlights the mechanisms underlying the regulatory role of PUM proteins and, most importantly, the emerging impact of PUM dysregulation in cancer. It also emphasizes the importance of upcoming studies on PUM proteins in the context of cancer, as they may provide new therapeutic targets in the future.

**Abstract:**

Until recently, post-transcriptional gene regulation (PTGR), in contrast to transcriptional regulation, was not extensively explored in cancer, even though it seems to be highly important. PUM proteins are well described in the PTGR of several organisms and contain the PUF RNA-binding domain that recognizes the UGUANAUA motif, located mostly in the 3′ untranslated region (3′UTR) of target mRNAs. Depending on the protein cofactors recruited by PUM proteins, target mRNAs are directed towards translation, repression, activation, degradation, or specific localization. Abnormal profiles of PUM expression have been shown in several types of cancer, in some of them being different for PUM1 and PUM2. This review summarizes the dysregulation of PUM1 and PUM2 expression in several cancer tissues. It also describes the regulatory mechanisms behind the activity of PUMs, including cooperation with microRNA and non-coding RNA machineries, as well as the alternative polyadenylation pathway. It also emphasizes the importance of future studies to gain a more complete picture of the role of PUM proteins in different types of cancer. Such studies may result in identification of novel targets for future cancer therapies.

## 1. Introduction

**Figure 1:**
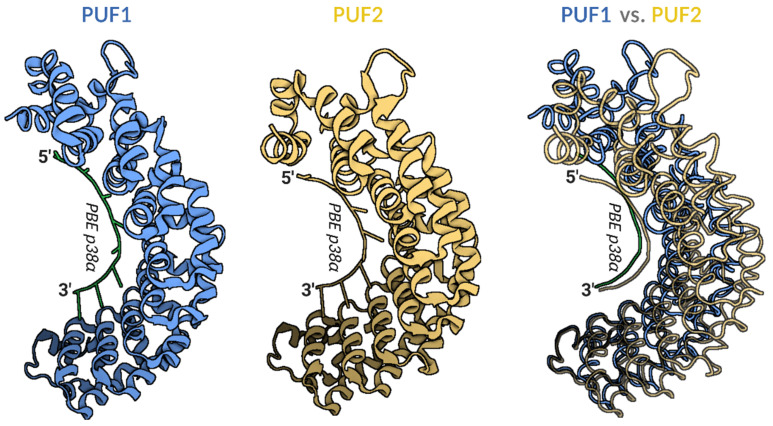
Models of human PUF1 and PUF2 RNA-binding domain crystal structure in complex with PUM-binding element (PBE) of p38α mRNA target. Left panel—cartoon model of PUF1 structure in a complex with PBE of p38α mRNA (PDB id:3Q0M); middle panel—cartoon model of PUF2 structure in a complex with PBE of p38α mRNA (PDB id:3Q0R); right panel—overlay of tube models of PUF1 (blue) and PUF2 (yellow) structures in complex with PBE of p38α mRNA. The overlay panel shows a more open PUF1 compared to the PUF2-domain, binding the same PBE of p38α mRNA. Visualized models were obtained using BioRender.com from 3Q0M and 3Q0R PDB structures [10]. PUF—PUM RNA-binding domain; PBE—PUM binding element UGUAHAUW; PDB—Protein Database Bank.

A large body of knowledge regarding genetic mutations and associated transcriptional dysregulation, which is critical for carcinogenesis, has been accumulated in recent decades. Although growing evidence indicates that dysregulation of post-transcriptional gene expression plays a highly important role in cancer as well, with the exception of microRNAs this role has been neglected. Post-transcriptional gene expression regulation (PTGR) mainly encompasses RNA processing, which takes place beyond transcription, starting in the nucleus and continuing in the cytoplasm [1]. The main nuclear RNA processing steps consist of 7-methylguanosine cap synthesis on the 5′-end of pre-mRNA, generation of the 3′-end by cleavage and polyadenylation, including alternative polyadenylation (APA), and a multistep splicing reaction that removes introns and produces mature mRNA molecules. These nuclear RNA processing events are mediated by a multitude of RNA-binding proteins (RBPs) that associate with the nascent mRNA as soon as the 5′-end is transcribed via RNA polymerase II catalysis [2]. As is now well-known, a variety of RBPs are dysregulated in different cancer types (for a review, see [3]). This review focuses on Pumilio proteins, PUM1 and PUM2, which represent well-described examples of RBPs playing a role in PTGR in metazoans, including humans (for a review, see [4]), as well as in plants [5]. There is accumulating evidence now that expression of PUM proteins is dysregulated in several cancers (for a review, see [4]). PUM proteins contain a C-terminal highly conserved PUF domain that binds to mRNA molecules. This domain consists of eight imperfect tandem repeats of 36 amino acids (aa) (Figure 1). These repeats form a curved structure, each of which is in contact with one base within a PUM-binding element (PBE)—an eight-nucleotide conserved motif UGUAHAUW, located mostly in the 3′ untranslated region (3′UTR) of target mRNAs (Figure 1) [6,7,8,9].

**Figure 2:**
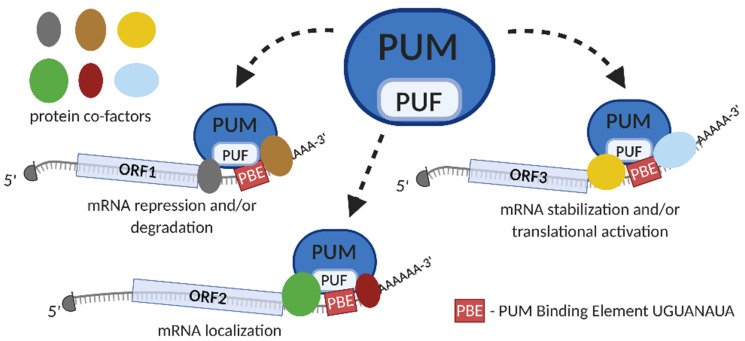
Model of Pumilio proteins (PUM)-mediated posttranscriptional gene regulation. PUM protein contains RNA-binding domain PUF (in pale blue). PUM protein binds to PBE motif (rectangle in red), located within 3′ untranslated region (3′UTR) of specific mRNA targets (1, 2 or 3), by engaging PUF domain. PUM-binding enables recruitment of different protein cofactors, presented schematically as ovals (in grey, brown, yellow, green, maroon and blue). Depending on the protein complexes that are formed upon PUM-binding, the mRNA target is directed towards repression and/or degradation, specific subcellular localization or stabilization and/or translational activation. The 5′cap structure is presented as the grey half circle; ORF—open reading frame.

While the original *Drosophila melanogaster* PUM protein is unique, there are several Pum proteins that exist in yeast, *Caenorhabditis elegans*, and other model organisms. There are two *PUM* genes in humans on chromosomes 1 and 2 called *PUM1* and *PUM2*, respectively, and they encode PUM1 and PUM2 proteins, respectively (for a review, see [4]). The rate of the overall aa similarity between PUM1 and PUM2 protein is 83%, with the PUF-domain being 91% identical [10]. PUMs bind to different protein cofactors, such as NANOS [11], DAZ-like (DAZL) [12], and DEAD-Box Helicase 20 (DDX20) [13]. Binding of PUM proteins with their co-factors in most cases directs a given mRNA towards repression, leading mostly to degradation. This binding, however, may also stabilize mRNA, activate translation, and/or storage in specific subcellular compartments (Figure 2) (for a review, see [4]). It has been demonstrated that mRNA repression by PUM proteins consists of the promotion of deadenylation [14,15,16], and in some cases, also decapping [17]. Deadenylation is followed mostly by degradation [18,19], which is in line with the finding that the presence of PBEs is closely associated with lower mRNA stability [20,21,22]. This phenomenon was confirmed by studies in several human cell lines, such as U2OS, HEK293, and HeLa [23,24,25]. The process of deadenylation is based on PUM interaction with components of the deadenylation Ccr4-Not (CNOT) complex [15,19,26]. Studies in *D. melanogaster* and humans indicate that PUM proteins also use an additional mechanism to repress their target mRNAs translation, namely antagonizing poly-A-binding protein (PABP) [27].

There is growing evidence that mammalian PUMs control stem cell fate in many contexts. For example, human PUM2 represses mRNA encoding ERK2 and p38α, homologues of mitogen-activated kinase (MAPK) in human embryonic stem cells, by directly binding their PBEs [28]. MAPK controls many aspects of development, such as cell proliferation, differentiation, and survival (for a review, see [29]). Therefore, PUM2, by repressing those mRNAs, may be involved in the control of stem cell fate as well as in tumor progression. However, these hypotheses require further study. Additionally, it was discovered that PUM1 is necessary for haploid mouse embryonic stem cells (mESCs) to exit self-renewal. It does so by repressing pluripotency transcription factors, thus promoting differentiation [30]. Likewise, in the diploid mESCs, PUM1 promotes differentiation, since mESCs lacking PUM1 showed increased expression of pluripotency markers but not the differentiation genes [31]. Interestingly, PUM2 protein turned out to play an opposite role, i.e., ESCs lacking PUM2 showed decreased levels of pluripotency markers, thus accelerating differentiation. Finally, both PUM1 and PUM2 are essential for mouse embryogenesis, since a double PUM1/2 knockout resulted in developmental delay and lethality at the morula stage [31]. A significant influence of PUM1 and a slightly smaller PUM2 on body size control was recently demonstrated in the mouse. It is at least partly based on PUM1/2-stimulated repression of mRNA encoding cyclin-dependent kinase (CDK) inhibitor CDKN1B, which is a regulator of the G1/S transition during the cell cycle. Posttranscriptional repression of CDKN1B by PUM1/2 enables the control of cell proliferation and acts as one of the crucial factors contributing to the body size determination of the growing embryo [32].

## 2. Expression of Pumilio Proteins (PUM1/2) and mRNA Target Identification in Cancer

**Figure 3:**
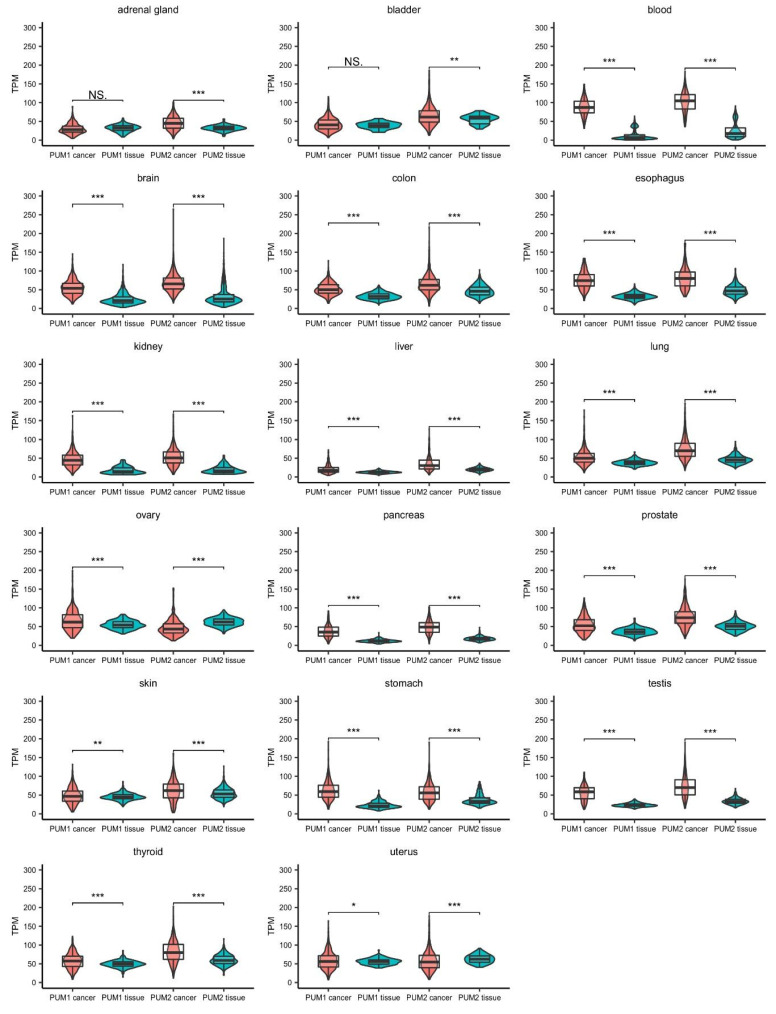
Comparison of PUM1 and PUM2 RNA expression levels in 17 types of cancers and corresponding healthy tissues. Data were obtained for cancers from The Cancer Genome Atlas (October 2020) (PUM1/2 cancer) and for healthy tissues from the Genotype-Tissue Expression project (October 2020) (PUM1/2 tissue). Statistical significance was calculated using *t*-tests. Violin-box-whisker plots were generated using ggplot2 library in R. NS—non-significant, TPM—transcript per million (reads), * *p* < 0.05, ** *p* < 0.01, *** *p* < 0.005.

*PUM* genes are ubiquitously expressed in human tissues. Taking advantage of the Cancer Genome Atlas database, we analyzed RNA expression levels of *PUM1* and *PUM2* from human cancer samples and compared them to *PUM* expression in healthy tissues collected within the Genotype-Tissue Expression (GTEx) project. The accumulated data show that the expression levels of *PUM1* and *PUM2* are significantly altered in 17 types of cancer tissues (Figure 3). Notably, in the majority of cancer tissues, PUM2 RNA expression was considerably higher than PUM1 expression. Interestingly, in almost all of the samples, the PUM1 level was increased compared to healthy tissues (except adrenal gland and bladder cancers). In the case of PUM2, it was overexpressed in almost all samples, except ovarian and uterus cancer tissues, where the RNA expression level was lower than in healthy tissues. These differences in expression patterns between PUM1 and PUM2 may indicate that they have divergent functions in adrenal gland, bladder, ovarian, and uterus cancers. Further studies are required for the proper interpretation of PUM1 and PUM2 expression patterns. In particular, alterations of RNA expression of PUMs require validation at the protein level.

To gain comprehensive insights into the importance of PUM-controlled PTGR in cancer as well as into potential functional differences between PUM proteins, the direct identification of PUM-bound RNAs by protein-RNA co-immunoprecipitation (co-IP) followed by RNA-Seq analysis was performed in TCam-2 cells. This cell line represents seminoma, exemplifying a type of testis germ cell tumor [33]. Considering that RNA-PUM binding does not imply regulation per se, transcriptome-wide identification of quantitative changes of mRNAs upon knock-down of PUM proteins was conducted in parallel. In this study, only mRNAs fulfilling two criteria were considered as regulated by PUM1 or PUM2 proteins, namely (1) binding to PUM, and (2) changing levels, as detected by differential gene expression analysis upon PUM knockdown. In total, 346 PUM1-regulated and 141 PUM2-regulated targets were identified in that study. About 90% of PUM-regulated targets were different for PUM1 and PUM2, and nearly 100% of all identified targets contained PBEs, thus validating the results [34]. Several other studies aimed at the global identification of human PUM1 and PUM2-regulated targets were performed in other human cell lines and using some methodological modifications compared to the study in TCam-2 cells. They all showed that the number of non-overlapping PUM1 and PUM2 targets was significant, ranging from 57% to 95% [35,36,37,38].

The high numbers of PUM1 and PUM2-specific targets that have been identified in those studies is puzzling, considering that despite the fact that PUF1 domain is less curved than PUF2 (Figure 1), these proteins recognize the same PBE motif. Notably, in contrast to the highly conserved C-terminal, the N-terminal regions of PUM1 and PUM2 exhibit a much lower degree of evolutionary conservation and contain low-complexity regions with insertions and deletions [39]. It was demonstrated that the N-terminal region of human PUM2 serves as an interface for interaction with other proteins, such as DDX20 (GEMIN3) [13]. Additionally, based on studies of the PUM cofactors—the NANOS family—it was demonstrated that only NANOS3 protein, and not NANOS1 or NANOS2, cooperates with PUM2 in repression of mRNA encoding the SIAH1 tumor suppressor [40]. This study showed that PUM may have different mRNA targets in cooperation with different combinations of cofactors. Therefore, it was proposed that PUM1 and PUM2 regulate separate mRNA pools by co-operating with different proteins. A global immunoprecipitation (IP) and mass spectrometry (MS)-based identification of PUM1- and PUM2-binding proteins showed that PUM1 and PUM2 indeed interact mainly with different groups of proteins, a majority of them representing RBPs. Combinatorial analysis of RNA immunoprecipitation (RIP) and RNA-Seq MS, as well as motif enrichment analysis for RNA-binding proteins in PUM1 and PUM2 mRNA targets, revealed that PUM1 and PUM2 form separate ribonucleoprotein networks [34], which resemble so-called “regulons” or “posttranscriptional operons”, as previously suggested for RBPs that recognize specific motifs in RNAs [41]. According to those networks, PUM1 and PUM2 may cooperate with varied protein cofactors to regulate mRNA target sub-pools responsible for unique pathways. For example, PUM1 with IGF2BP3 cofactor may co-regulate mRNAs implicated in regulation of cell division, while PUM1 with PABPC4 and MATR3 may co-regulate mRNAs involved in the epidermal growth factor receptor-signaling pathway. On the other hand, PUM2 with G3BP2, HNRNPA1, FXR2, and SFPQ co-factors may co-regulate mRNAs involved in the regulation of Rho protein signal transduction, while with MATR3, PUM2 may affect mRNAs that negatively regulate cell development [34]; however, such scenarios require validation at the protein level. One important aspect that should be taken into account when discussing the functional relationship between PUM1 and PUM2 interactomes is that PUM1 and PUM2 mRNAs contain functional PBEs in their 3′UTRs. Consequently, as has already been shown at the RNA and protein levels, they repress each other [42,43].

## 3. Implication of PUM Proteins in Specific Types of Cancer

In addition to the regulation of stem cell fate, growth, and development, PUMs are involved in the control of a myriad of other biological functions such as hematopoiesis, neurogenesis, and gametogenesis, while their dysfunction contributes to several diseases, which is in line with their ubiquitous pattern of expression, as they have been identified in many human tissues.

Notably, there is an emerging field of research concerning the involvement of PUM proteins in specific types of cancer. It has also been demonstrated that PUM proteins control the levels of several mRNAs that encode proteins involved in processes that are often disrupted in cancerogenesis, such as apoptosis, proliferation, and the cell cycle.

### 3.1. PUM Proteins in Leukemia

Hematopoiesis is the process of differentiation of hematopoietic stem cells (HSCs) residing in the bone marrow, giving rise to various types of blood cell. Leukemia occurs when cancer-driving mutations cause conversion of some HSCs to leukemia stem cells (LSCs), which then abnormally proliferate and grow in size, resulting in bone marrow malfunction. New evidence suggests that PUM1 and PUM2 play vital regulatory roles in the maintenance and proliferation of normal human and mouse stem cells (SCs) [44,45]. It has also been demonstrated that both PUMs are overexpressed in a majority of acute myeloid leukemia (AML) samples, as well as in the cell-lines derived from those pathogenic samples [44]. Importantly, PUM1 and PUM2 influence the cell cycle, proliferation, and apoptosis of normal human and mouse HSCs, as well as AML cells [44]. PUMs induce these effects by activating the expression of Forkhead box protein P1 (FOXP1) transcription factor mediated by direct binding PBE motifs located in the 3′UTR of FOXP1 mRNA. This PUM-mediated FOXP1 activation suppresses the expression of cell cycle inhibitors, such as CDKN1B, thereby promoting proliferation. This is in line with the findings that FOXP1 itself regulates cell proliferation and differentiation and is crucial for the regulation of hematopoietic stem progenitor cells during leukemic cell growth [44]. Importantly, PUM1/2 can also directly suppress the expression of a tumor suppressor, CDKN1B [24,32,43], potentially enhancing the FOXP1 pathway in HSCs and AML cells. Additionally, the expression levels of multiple other proteins involved in HSCs and LSCs are likely to be influenced by PUM1/2 3′UTR-mediated regulation, which is a topic for future study [44].

### 3.2. PUM Proteins in Seminoma Testis Germ Cell Tumor

Seminoma is the most common type of testis germ cell tumor among western populations and is increasing in frequency, with the highest incidence in men at reproductive age [33]. In the TCam-2 seminoma cell line, it has recently been demonstrated that PUM1 and PUM2 regulate several mRNAs functionally linked to cancer. Among such PUM1 and PUM2-repressed targets, mRNA encoding SPINDLIN1 (SPIN1) is known for its role in mammalian gametogenesis. This spindle-binding protein is necessary for the meiotic progression of germ cells, while abnormal overexpression of SPIN1 is associated with human ovarian cancers and has been observed in some other cancer cell lines [18,46,47,48]. SPIN1 protein is expressed in TCam-2 cells and its overexpression causes a significant increase in proliferation and a reduced level of apoptosis. Those two features indicate that SPIN1 plays a proto-oncogenic role in TCam-2 cells. PUM1 and PUM2 repress a SPIN1 homologue called SPIN3, whereas its overexpression elicits a decrease in proliferation and an increase in apoptosis of TCam-2 cells [42]. Taken together, SPIN1 demonstrated proto-oncogenic properties, while SPIN3 showed tumor suppressor features. PUM1 itself, but not PUM2, strongly stimulated apoptosis and moderately slowed down cell cycle progression, suggesting that PUM1, similarly to SPIN3, plays the role of a tumor suppressor in TCam-2 cells. Altogether, by acting as SPIN1 and SPIN3 repressors, PUM proteins may promote a normal human male germ cell apoptotic status and thus prevent cancer [42].

In TCam-2 cells, PUMs also cause repression of mRNA encoding kinesin KIF18A [49]. This kinesin exists on positively charged ends of microtubules in the vicinity of the kinetochore, and it regulates the dynamics of mitotic cell division. The knockout of Kif18a in a mouse causes mitotic arrest and apoptosis of the male germ cells, resulting in infertility. This kinesin is an important regulator of the cell cycle and apoptosis in human germ cells as well [50,51]. Notably, it also plays the role of a proto-oncogene, as it is overexpressed in many cancer types. It has been reported that KIF18A positively influences TCam-2 cell proliferation, downregulates apoptosis, and promotes cell cycle progression, these effects being opposite to the effects of PUMs. Therefore, repression by PUM proteins may represent one of the mechanisms affecting KIF18A levels in regulating proliferation, the cell cycle, and apoptosis in TCam-2 cells [49].

The findings concerning PUM1 and PUM2 mRNA targets and their functional relations in TCam-2 cells are of interest and should be validated in patients suffering from testis germ cell tumors and several types of cancer affecting other human tissues.

## 4. Interplay between PUM and Non-Coding RNAs in Cancer

The functional relationship between 3′UTR-binding RBPs and non-coding RNAs is an emerging topic in PTGR research. Until recently, this issue had not been sufficiently explored due to methodological limitations. Several studies on the mechanisms of the interplay between PUM and non-coding RNAs have been published in recent years. These studies provide valuable data on the interplay between PUM and non-coding RNA in the context of cancer.

### 4.1. PUM Proteins Cooperate with microRNA Machinery in Cancer

First, in global screens of HeLa cells for PUM mRNA targets, it was documented that microRNA complementary sites were enriched in the vicinity of PBE motifs within PUM target mRNAs [35]. The significance of that vicinity has partly been clarified in studies describing the repression of mRNA encoding cyclin-dependent kinase inhibitor 1B (CDKN1B) by PUM1/2 proteins [43]. CDKN1B downregulates cell cycle progression by blocking CDK2 activity. It plays the role of a tumor suppressor, its level being lowered in several types of cancer, while becoming increased in non-dividing cells [52]. CDKN1B mRNA is a target for miR-221- and miR-222-mediated repression, and high levels of these two miRNAs correlate inversely with low CDKN1B levels in cancer, for example in glioblastoma [53]. It was shown in human fibroblasts that PUM binding to 3′UTR of CDKNB1 mRNA induces a local conformational rearrangement, making the miRNA complementary site accessible for miRNA hybridization. Through that mechanism, PUM1/2 enables the miRNA-induced repression of CDKN1B mRNA translation [43].

Cooperation between the repression by PUM proteins and miRNAs-induced repression was also demonstrated in the regulation of E2F3 transcription activator, a well-described human oncogene. E2F3 belongs to a family of E2F transcription factors, which regulate cell proliferation as well as apoptosis, while their dysregulation frequently accompanies human malignancies [54]. The 3′UTR of mRNA encoding E2F3 contains two PBE motifs, which have been shown to be active in PUM-induced repression, as well as several complementary sites for miRNAs. It has been demonstrated that PUM1/2 proteins downregulate E2F3 expression cooperatively with mir-503 and mir-125pp. Interestingly, several cancer cell lines escape the PUM-mediated regulation by shortening the E2F3 3′UTR, eliminating PBEs, and thus disabling PUM-mediated repression [55]. In some cancer types, e.g., in bladder carcinoma, mir-503, and mir-125p, levels are significantly decreased, indicating their importance and their anti-oncogenic role [56].

### 4.2. Long Non-Coding RNA Sponge Activity in PUM Sequestration and Genome Stability

Long non-coding RNAs (lncRNAs) are molecules longer than 200 nucleotides and lack an open reading frame. lncRNAs often contain microRNA complementary sites and act as miRNA sponges, thereby regulating their levels inside the cell. This feature of lncRNAs may result in upregulation of several oncogenic miRNA targets, thus contributing to carcinogenesis [57,58]. Interestingly, two simultaneous studies showed that a lncRNA, known as non-coding RNA activated by DNA damage (NORAD), contains a number of motifs that correspond to several RBPs, including 15 PUM-corresponding PBEs [23,37], suggesting a sponge-activity-based PUM sequestration. Given the diverse roles of PUM proteins in regulating proliferation, the cell cycle, and apoptosis-related mRNAs, as discussed in the previous sections, the regulation of PUM activity inside the cells by NORAD-mediated sequestration exerts a significant impact on carcinogenesis. NORAD is a ubiquitous, abundant, and conserved lncRNA in mammals. Both PUM1 and PUM2 bind to NORAD lncRNA. Compared to PUM1, PUM2 has a higher affinity for NORAD. The identification of PUM2 mRNA targets performed upon NORAD knockdown demonstrated the activity of the NORAD–PUM interaction and its importance for fine-tuning the levels of mRNAs encoding proteins related to DNA repair and the cell cycle [37]. Importantly, inactivation of NORAD in the HTC116 cell line leads to chromosomal instability by increasing chromosomal and mitotic abnormalities. These studies brought attention to the role of the NORAD–PUM interaction in regulating genomic stability. Mass spectrometry analysis identified over 500 RBPs that bind to NORAD fragments in the U2OS cell line. Interestingly, one of the identified RBPs is SAM68, a protein required for PUM activity regulation by NORAD. It was additionally demonstrated that SAM68 and PUM2 interact independently of NORAD, an interaction that is required for PUM2 binding to NORAD [59].

Further research aimed at elucidating the exact molecular mechanism of the control of genome stability by the NORAD–PUM axis showed opposing findings. Previous findings showed that NORAD is predominantly located in the cytoplasm and acts as a PUM sponge, but single-molecule RNA fluorescent in situ hybridization (smRNA FISH) demonstrated that NORAD is also located in the nucleus. In addition, upon stress, NORAD localization in the nucleus substantially increases. Finally, NORAD was shown to regulate genomic stability by modulating the availability of the RNA Binding Motif Protein X-Linked (RBMX) protein inside the nucleus for topoisomerase complex formation, and PUM was shown to be dispensable for NORAD function [60]. A follow-up study addressed these findings and showed that NORAD indeed acts as a PUM sponge inside the cytoplasm, independently of stress, and regulates genomic stability and proper mitotic divisions, although PUM sequestration and RBMX are dispensable for this function [61]. The question of physiological significance of the NORAD–PUM interaction was addressed by using a mouse model. The research demonstrated that removal of mouse NORAD lncRNA, having 61% sequence identity to human NORAD, resulted in a phenotype that resembles premature aging due to genomic instability and mitochondrial dysfunction [62]. PUM2 overexpression resulted in a phenotype similar to NORAD-deficient mice.

Although there are opposing results regarding NORAD localization, its role in regulating RBMX protein availability for topoisomerase complex formation, and whether this is required for NORAD function in genomic stability, the clear evidence presented by multiple studies shows that NORAD is a conserved lncRNA that regulates PUM proteins in mammals [60]. This regulation is critical for genomic stability and proper mitotic division. These studies served as a proof that lncRNAs function as RBP sponges inside the cells and, most importantly, they demonstrated the critical role of the PUM sequester by NORAD in carcinogenesis.

## 5. PUM-Mediated Repression and Alternative Polyadenylation in Cancer

Alternative polyadenylation (APA) is a mechanism of gene expression regulation that uses canonical or non-canonical alternative polyadenylation sites. This generates alternative 3′ ends of the transcripts synthesized by polymerase II, thereby creating shorter or longer 3′UTRs. APA strongly impacts PTGR by removing or retaining regulatory elements, such as RBP- or microRNA-interaction sites, which control the fate of mRNA [63]. Notably, APA strongly contributes to tumorigenesis [64], and hence the prognosis for APA-positive patients in several types of cancer is poor [65,66,67]. A global APA analysis of the transcriptome of triple-negative breast tumors (TNBTs) demonstrated that it is a widespread phenomenon. In that model, shortening of 3′UTRs causes significant PBE motif removal compared to normal breast tissue [68], as was previously demonstrated for E2F3 mRNA [55]. The PBE removal from 3′UTRs caused an increased level of Phosphatase and tensin homolog (PTEN), Neuroblastoma RAS (NRAS), and FOXO1 proteins, and their corresponding mRNAs contained shortened 3′UTR. Based on the aforementioned observations, a question was raised, namely why mRNAs containing PUM-binding sites are more frequently affected by APA compared to other mRNAs. This issue was at least partly clarified by showing that two main polyadenylation complexes, namely the cleavage factor I (CFI) complex and the cleavage stimulation factor (CSTF) complex, recognize the GU-rich sequences that resemble PBE motifs. Specifically, the CFI complex that mediates transcript cleavage binds upstream from the poly-A site and interacts with PBE PUM motif UGUA [65,69] and UGUAXAUA [35,68]. The CSTF complex binds downstream of the poly-A site to another GU-rich region [69], which also represents fragments of the PBE motif [68]. Interestingly, upregulation of the CSTF3 polyadenylation complex is common in TNBT cancer cell lines, and this upregulation induces APA of two mRNAs encoding crucial tumorigenesis players, NRAS and c-JUN. In both transcripts, PBE motifs are absent in their corresponding shortened 3′UTRs, resulting in translational upregulation of both proteins as well as their downstream targets. Based on this data the following scenario has been proposed: CSTF3 upregulation enables preferential usage of motifs that are recognized by PUM [68]. Therefore, APA of PUM targets may contribute to TNBT phenotypes.

## 6. Conclusions and Perspectives

Although it has become clear that PUM proteins are important players in human health and disease, their role in cancer has not been sufficiently explored, and several important issues should be addressed. First, it is unclear whether *PUM* gene mutations in the patients underlie at least some types of cancer. Such research has not been reported so far but seems crucial to assess the role of PUM proteins in tumorigenesis. Second, it is vital to explore the mechanisms of PUM expression at the transcriptional as well as posttranscriptional levels in normal tissues, and dysregulation of those mechanisms in different types of cancer. Third, it must be explained how such dysregulation affects the structure and dynamics of PUM1 and PUM2 interactomes in the course of carcinogenesis. Finally, it is necessary to assess the impact of PUM dysregulation on the cell cycle in cancer tissues and on processes that are often disturbed in cancer, such as proliferation and apoptosis. Such studies would be valuable since they may provide new candidate targets for future therapies.

## Data Availability

Not applicable.
